# Supplementary material for: G-quadruplex DNA drives genomic instability and represents a targetable molecular abnormality in ATRX-deficient malignant glioma
Source: Nat Commun. 2019 Feb 26;10:943. doi: 10.1038/s41467-019-08905-8 (PMC6391399; doi:10.1038/s41467-019-08905-8)
Supplement: Supplementary file 1 — Supplementary Information [file 41467_2019_8905_MOESM1_ESM.pdf]

G-quadruplex DNA drives genomic instability and represents a targetable molecular abnormality in ATRX-deficient malignant glioma

Wang Y et al.

Supplementary Information

<u>Contents</u>

Supplementary Tables: pp. 2-3

Supplementary Figures: pp. 4-16

Target	Merchant, catalog number	Application
ATRX	Santa Cruz (H-300): sc-15408	Western Blots (1:500), IF (1:100)
VINCULIN	Sigma Aldrich (hVIN-1), V9131	Western Blots (1:5000)
phospho-KAP1 Ser 824	Abcam, ab70369	Western Blots (1;1000)
phospho-CHK1 Ser 317	Cell Signaling, 2344	Western Blots (1:500)
phospho-CHK1 Ser 345	Cell Signaling, 2348	Western Blots (1:500)
G-quadruplex, 1H6	Gift from Dr. Peter Lansdorp	IF (1:100)
G-quadruplex, BG4	Millipore, MABE917	IF (1:100)
γ-H2AX	Millipore (JBW301), 05-636	IF (1:500), ChIP (2 µg), IHC (Fr) (1:500)
53BP1	Novus Biologics, NB100-304	IF (1:1000)
Ki-67	Santa Cruz, sc-23900	IHC (P) (1:100)
BLM	Bethyl laboratories, A300-110A	IF (1:100), ChIP (5 µg)

Supplementary Table 1: Commercially available antibodies used in this study.

shCon-1	ATCTCGCTTGGGCGAGAGTAAG
shATRX-1	GGAACTAGCTCTTCAGAAA
shCon-2	CAACAAGATGAAGAGCACCAA
shATRX-2	GGAAAGATGATAAAGGAAA
shSCR	CAACAAGATGAAGAGCACCAA
sh590	CGACAGAAACTAACCCTGTAA

Supplementary Table 2: sequences of shRNA against ATRX

Tel 1/2 - Tel 1	GGTTTTTGAGGGTGAGGGTGAGGGTGAGGGTGAGGGT
Tel 1/2 - Tel 2	TCCCGACTATCCCTATCCCTATCCCTATCCCTATCCCTA
Tel 2-F	CAAGTTTAAGGTTGTGTTTGTCAC
Tel 2-R	AAATGAGTTGCAACAGGTACAAT
Tel X-F	TGTCTGGGTCTTTGGAGAGG
Tel X-R	CCTAACCCATCTGCTGGTTC
GAPDH ChIP-F	CGGGATTGTCTGCCCTAATTAT
GAPDH ChIP-R	GCACGGAAGGTCACGATGT
Myc-F	AGGGCTTCTCAGAGGCTTG
Myc-R	GCTGGAATTACTACAGCGAGTT
ZNF618-F	CGACGCCACCTAGAGGATAC
ZNF618-R	AATCTCTTACCCCTCCACTGC
ESR1-F	GCAGATCCAAGCTGTCTTTACTCA
ESR1-R	GGTGGGCAGAAGAAATCCTTT

Supplementary Table 3: sequences of primers used in this study

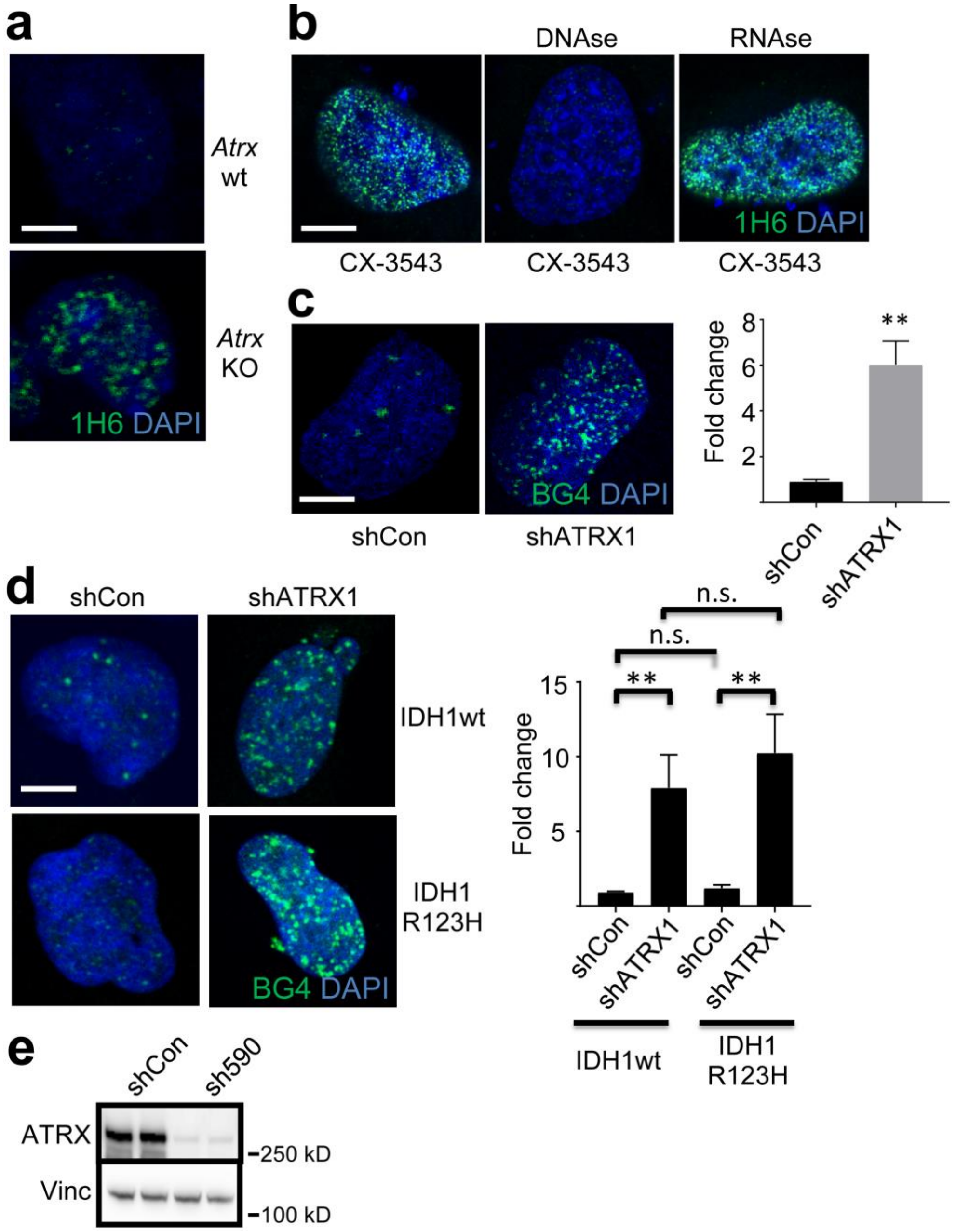

Supplementary FIG. 1: ATRX deficiency promotes G4 formation. a: G4 immunostaining (1H6) in primary murine neural progenitor cells harboring either intact or inactivated *Atrx*[44]. **b:** NHAs treated with 50nM CX-3543 alone (left), or with either DNase (middle), or RNase (right) to confirm the specificity of the 1H6 antibody for DNA structures. **c**: G4 immunostaining (BG4) in constitutive shATRX NHA lines (DAPI counterstain); quantified relative G4 signal intensity also shown (50 nuclei counted in all cases). **d**: G4 immunostaining (BG4) in constitutive shATRX NHA lines expressing either wild type (IDHwt) or R132H-mutant (IDH1 R132H) IDH1 (DAPI counterstain). Scale bars represent 10 µm; quantified relative G4 signal intensity also shown (50 nuclei counted in all cases). **e**: Western blot confirms robust ATRX knockdown in sh590-expressing TS 543 GSCs relative to controls (shCon, Vinculin control, duplicate loading). Where

applicable, error bars reflect SEM; *P* values determined by unpaired, two-tailed t-test (**: $P < 0.01$, n.s.: $P > 0.05$).

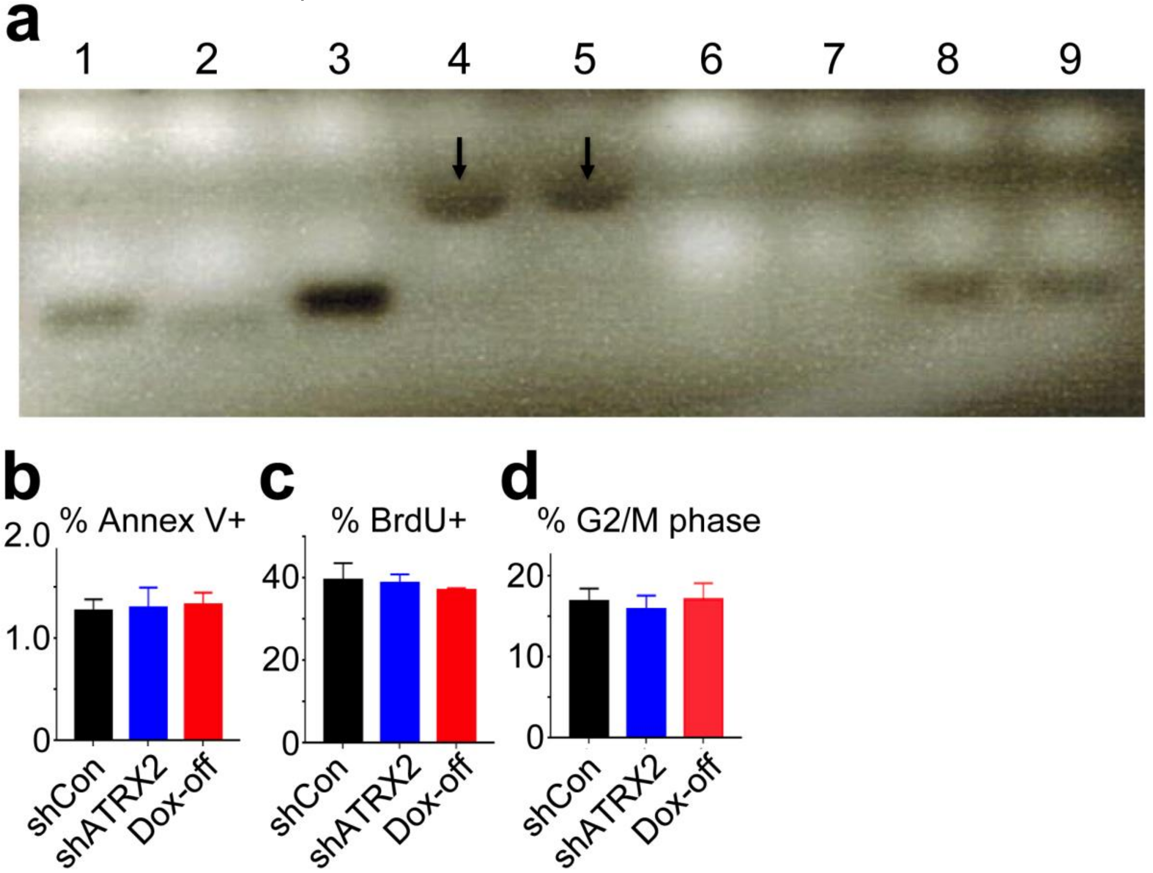

Supplementary FIG. 2: a: Confirmation of specificity for Hf2 G4 pulldowns using kit2 G4 nucleotides. Hf2 antibodies were incubated with kit2+random ssDNA mixture, no elution (lane 4), kit2+random dsDNA, no elution (lane 5), ssDNA alone (lane 6), dsDNA alone (lane 7), kit2+ssDNA, eluted (lane 8) and kit2+dsDNA, eluted (lane 9). Kit2 (lane 1), ssDNA (lane 2) and dsDNA (lane 3) were also included in the electrophoresis. Samples were run on a 3% agarose gel, non-denaturing. Arrows showed gel shift due to binding of pulldown antibodies. **b-d:** Inducible NHA lines (3 replicates each) showed no effects of ATRX inactivation on either apoptosis (measured by Annexin V positive population, **b**) or proliferation (measured by BrdU incorporation, **c**; G2/M-phase content (4n), **d**). Error bars reflect SEM; scale bars represent 10 µm.

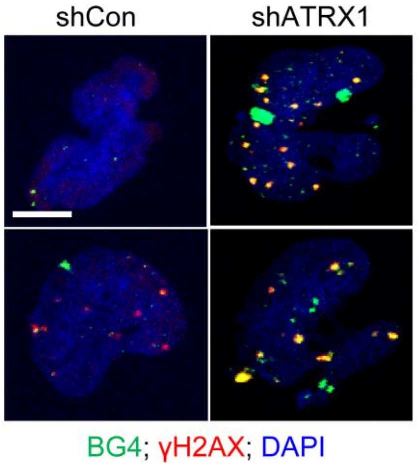

Supplementary FIG. 3: G4 and DNA damage sites colocalize in the setting of ATRX deficiency. Constitutive shATRX NHAs were double stained for G4 (BG4) and γ-H2AX. Two examples are shown for shCon and shATRX1 NHAs (DAPI counterstain). Scale bars represent 10 µm.

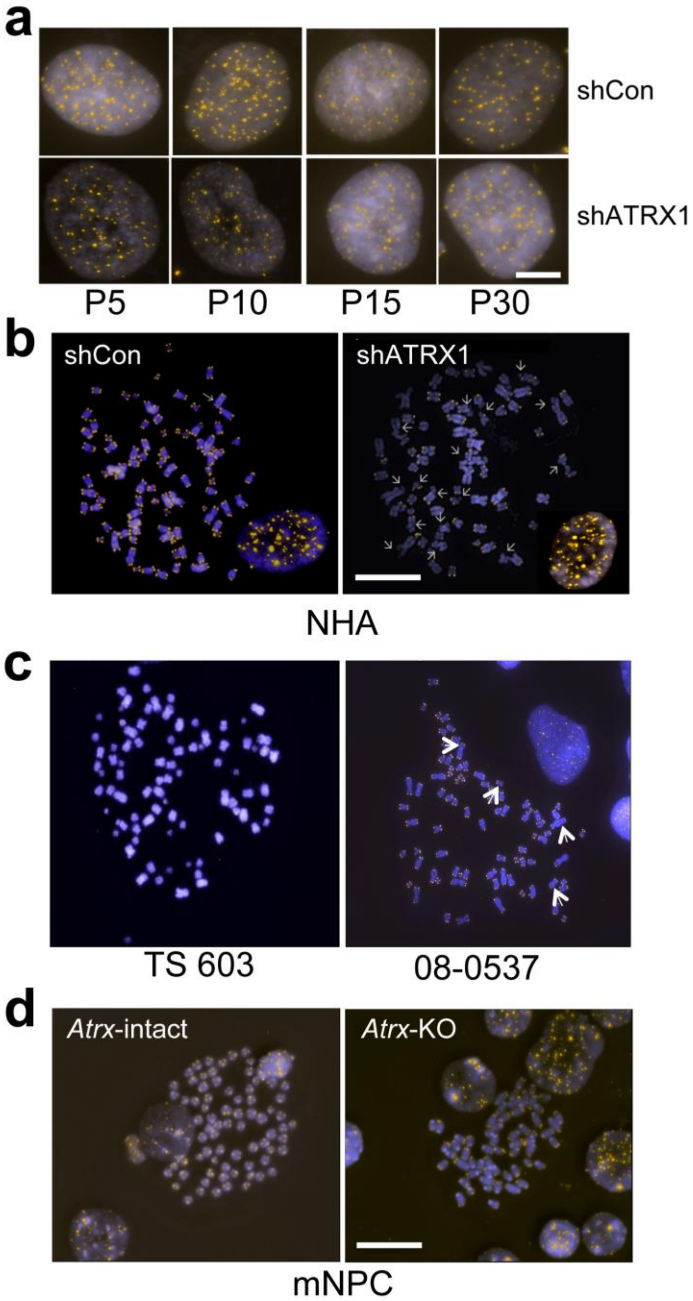

Supplementary FIG. 4: ATRX knockdown induces chromosome breaks, but not ALT in NHAs. a: TEL-FISH of NHAs at passages 5, 10, 15 and 30 after ATRX knockdown. No ultrabright foci, normally indicative of ALT, are apparent. **b:** ATRX deficient NHAs (shATRX1, passage 15) showed significantly increased chromosome breaks by cytogenetic analysis (white arrows). Despite these chromosome abnormalities, Tel-FISH (yellow) showed no change in telomere signal (Scale bar: 10 µm). **c-d**: cytogenetic analysis for chromosome breaks in *ATRX*-wild type (TS 603) and *ATRX*-mutant (08-0537) GSCs (c) and Tel-FISH in *Atrx*-intact and *Atrx*-KO mNPCs (d; Scale bar: 25 µm; white arrows indicate chromosome breaks in field of view).

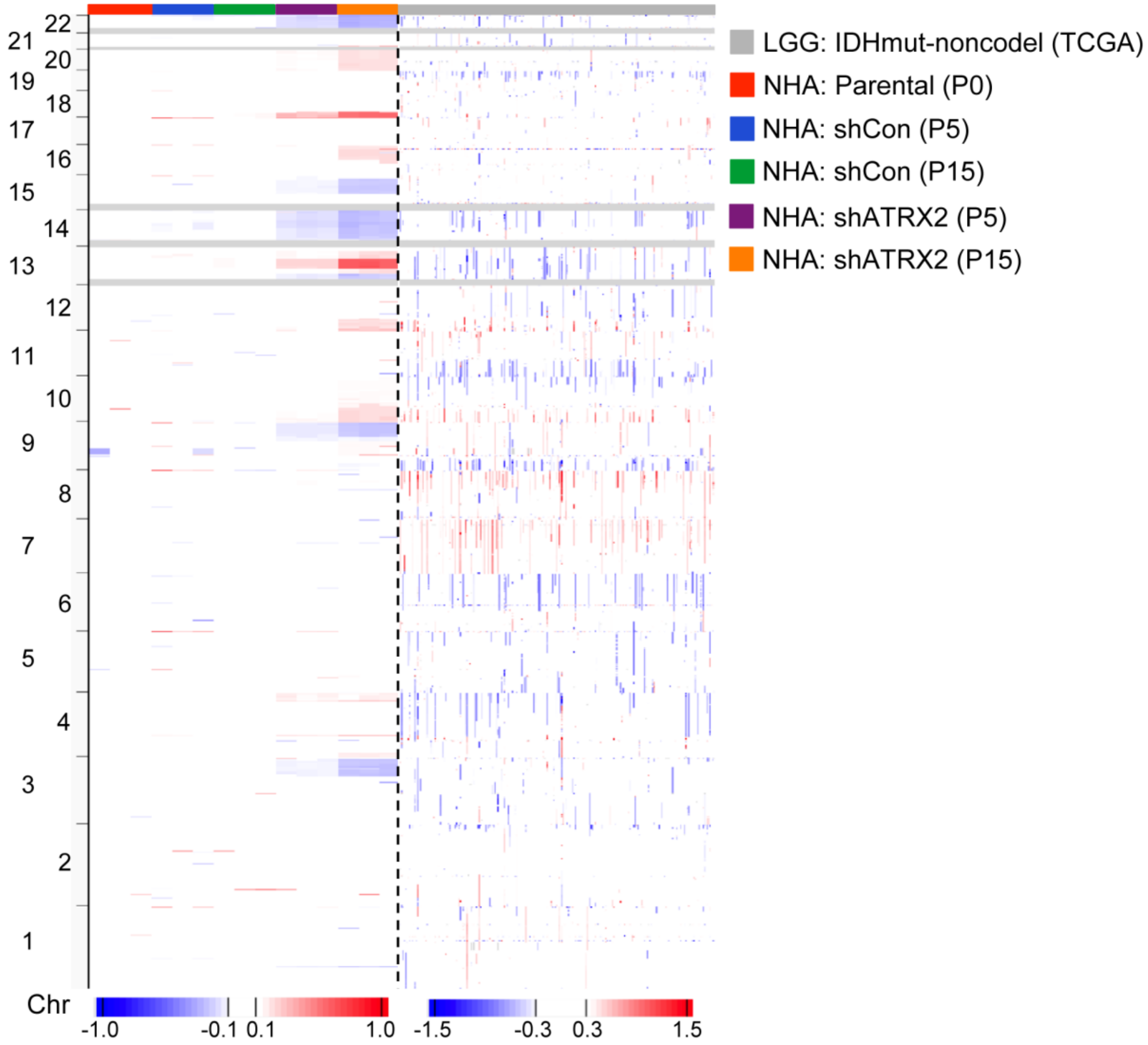

Supplementary FIG. 5: ATRX deficiency induces CNAs in NHAs and is associated with a distinct CNA profile in gliomas. Segmented SNP array data for parental NHAs, shCon NHAs (P5 and P15), shATRX1 NHAs (P5 and p15) and IDHmut-noncodel LGG patients (TCGA) visualized by IGV. Color scales indicate $\log_2$ copy number.

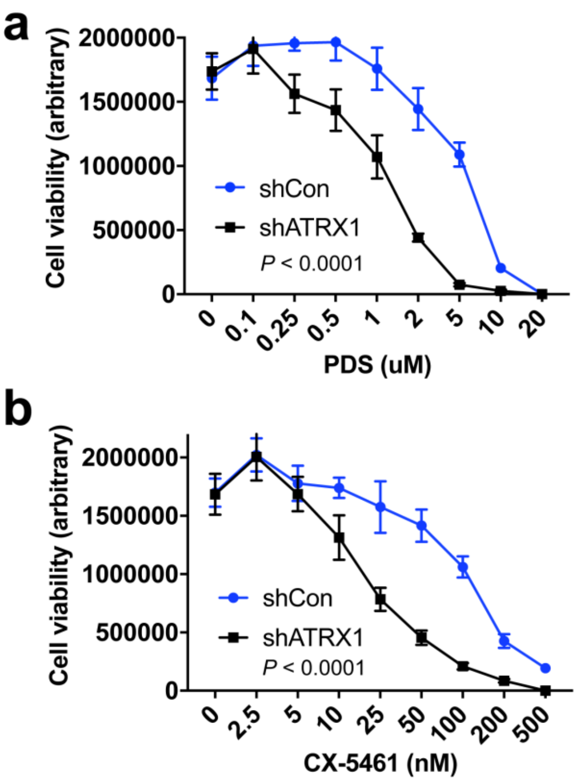

Supplementary FIG. 6: ATRX-deficient NHAs are selectively sensitive to G4 stabilization. a-b: Cell viability (CellTiter-Glo) of constitutive shCon and shATRX1 NHAs (3 replicates each) treated with either PDS (**a**) or CX-5461 (**b**) at indicated concentrations. Error bars reflect SEM; *P* values determined by two-way ANOVA.

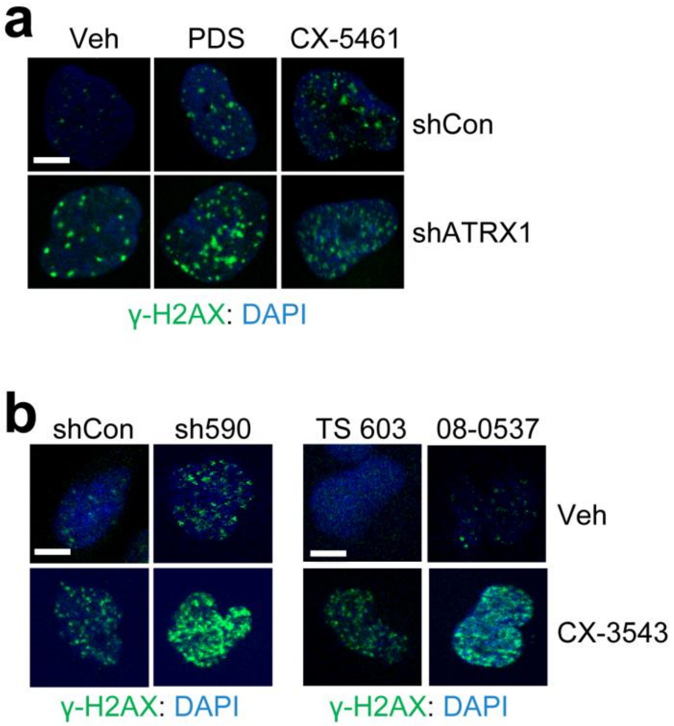

Supplementary FIG. 7: G4 stabilization markedly enhances DNA damage in the ATRX-deficient context. a: γ-H2AX immunofluorescence in constitutive shATRX NHAs showing increased DNA damage with either PDS (2 µM) or CX-5461 (50 nM) treatment, particularly in the setting of ATRX knockdown (DAPI counterstain). **b**: γ-H2AX immunofluorescence in ATRX-intact (shCon) and ATRX-knockdown (sh590) TS543 GSCs and in *ATRX*-wild type (TS 603) and *ATRX*-mutant (08-0537) GSCs treated with 100 nM CX-3543. Scale bars represent 10 µm.

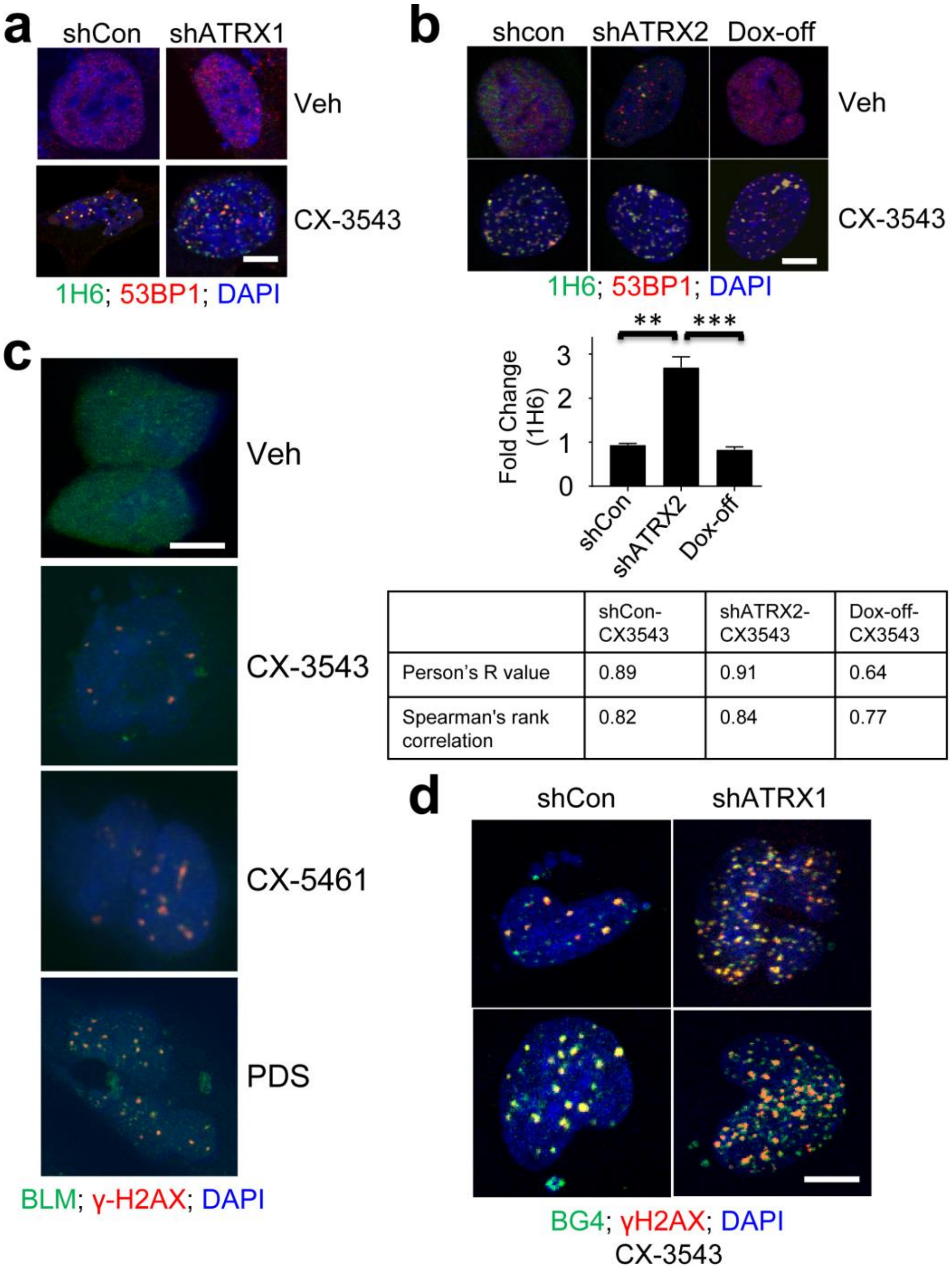

	shCon-CX3543	shATRX2-CX3543	Dox-off-CX3543
Person's R value	0.89	0.91	0.64
Spearman's rank correlation	0.82	0.84	0.77

Supplementary FIG. 8: G4s colocalize with DNA damage foci induced by CX-3543. G4 and 53BP1 coimmunofluorescence of constitutive (**a**) and inducible (**b**) shATRX NHAs treated with 100 nM CX-3543 (DAPI counterstain); quantified relative G4 signal intensity shown for inducible NHAs (50 nuclei counted in all cases), along with coefficients for spatial correlations between 1H6 and 53BP1 IF. **c**: Coimmunofluorescence for BLM and γ-H2AX reveals extensive colocalization in shATRX1 NHAs treated with either CX-3543 (100 nM), CX-5461 (50 nM), and PDS (2 µM). **d**: Coimmunofluorescence for G4 (BG4) and γ-H2AX in shATRX1 NHAs treated with 100 nM CX-3543. Two examples shown for treated and untreated cells. Scale bars represent 10 µm. *P* values determined by unpaired two-tailed t-test (**: $P < 0.01$, ***: $P < 0.001$).

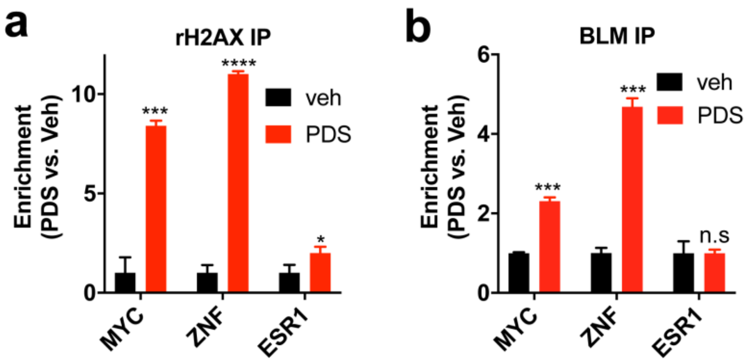

Supplementary FIG. 9: G4 stabilization enhances DNA damage at putative G4 sites. γ-H2AX (**a**) and BLM (**b**) ChIP in shATRX1 NHAs (3 replicates each) reveals highly significant qPCR enrichment at putative G4 sites in the promoter regions of *MYC* and *ZNF618* (ZNF). Enrichment at a control site (ESR1) is either modest (γ-H2AX) or not significant (BLM). Error bars reflect SEM; *P* values determined by unpaired two-tailed t-test (*: $P < 0.05$, ***: $P < 0.001$, ****: $P < 0.0001$, n.s.: $P > 0.05$).

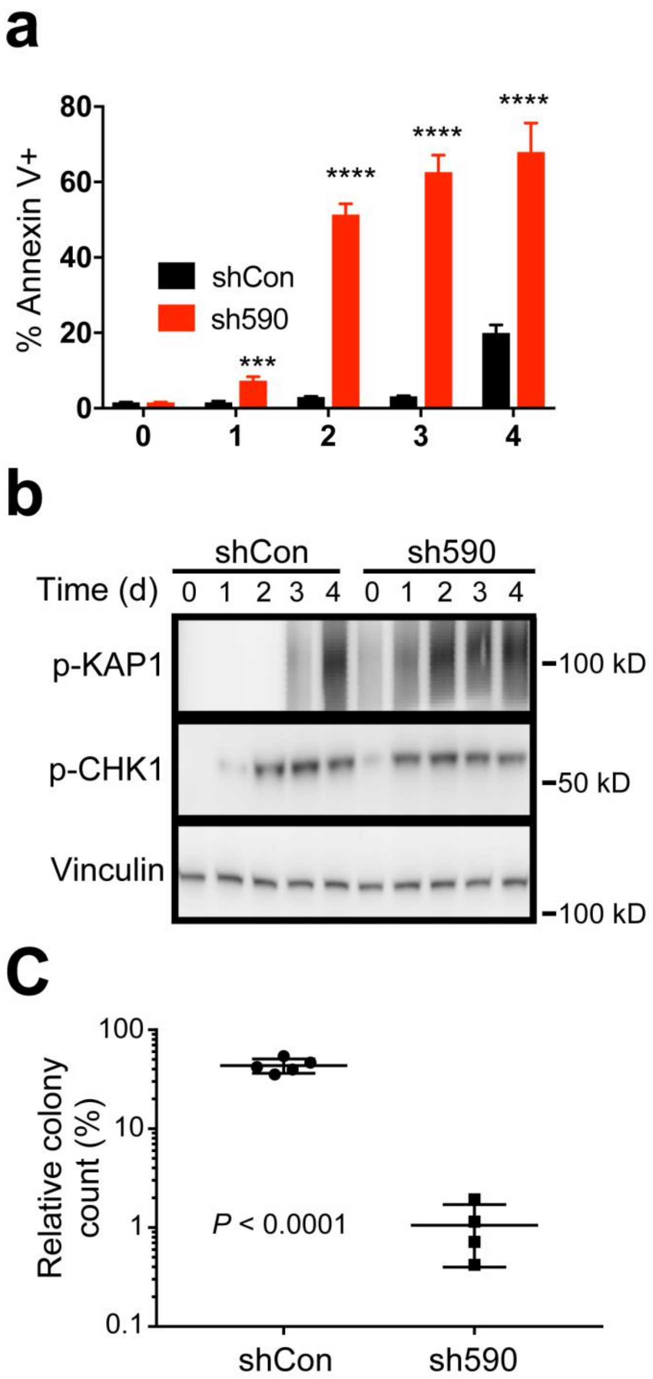

Supplementary FIG. 10: G4 stabilization selectively promotes replication stress and apoptosis and in ATRX-deficient GSCs. a-b: : Time course study in ATRX-intact (shCon) and ATRX-knockdown (sh590) TS 543 GSCs (4 replicates each) treated with 100 nM CX-3543 showing parallel kinetics of apoptosis (Annexin V positivity; a) and p-CHK1/p-KAP1 levels by western blot (b). **c:** ATRX-intact (shCon) and ATRX-knockdown (sh590) TS 543 GSCs (at least 4 replicates each) were subjected to soft agar colony formation assay and treated with radiation (2 Gy) and CX-3543 (50 nM). Colony number was quantified at 21 days, normalized relative to vehicle treated cells also subjected to 2 Gy, and compared to the number of colonies in unirradiated wells. Error bars reflect SEM; *P* values determined by unpaired two-tailed t-test (***: *P* < 0.001, ****: *P* < 0.0001).

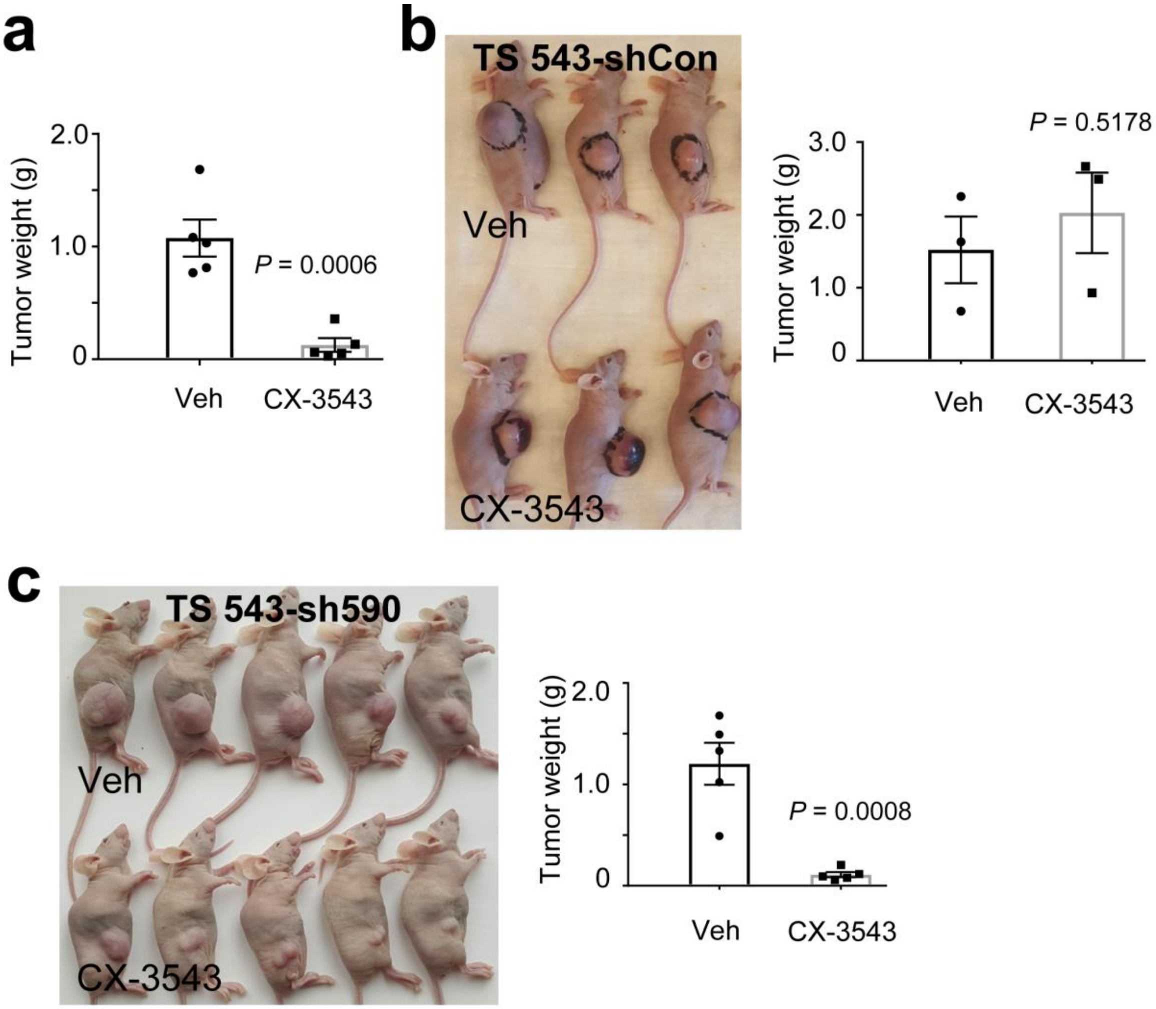

Supplementary FIG. 11: CX-3543 markedly slows the growth of ATRX-mutant glioma xenografts *in vivo*. a: Tumor weights of JHH-273 xenografts at study endpoint (See Materials and Methods) following treatment with either vehicle control (Veh) or 12.5 mg/kg CX-3543 (5 mice in each group). **b:** Representative image of mice bearing TS 543 (ATRX intact) xenografts following treatment with either vehicle (veh) or 12.5 mg/kg CX-3543 for 24 days. Tumor weights of TS 543-shCon xenografts at study endpoint (See Materials and Methods) following treatment with either vehicle control (Veh) or 12.5 mg/kg CX-3543 are also shown (3 mice in each group). **c:** Representative image of mice bearing TS 543 (sh590-ATRX knockdown) xenografts following treatment with either vehicle (veh) or 12.5 mg/kg CX-3543 for 19 days. Tumor weights of TS 543-sh590 xenografts at study endpoint (See Materials and Methods) following treatment with either vehicle control (Veh) or 12.5 mg/kg CX-3543 are also shown (5 mice in each group). Error bars reflect SEM; *P* values determined by unpaired two-tailed t-test.

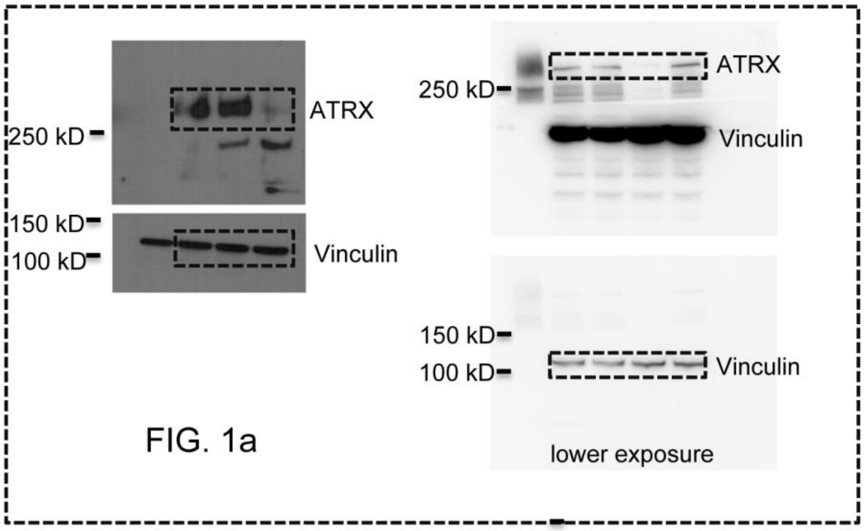

FIG. 1a

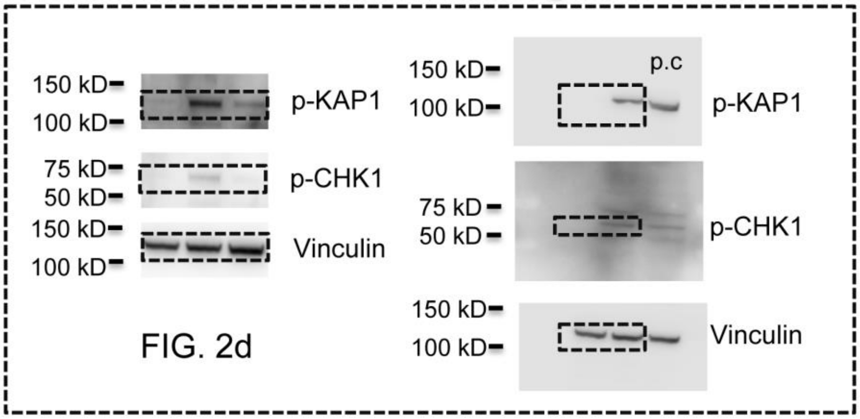

FIG. 2d

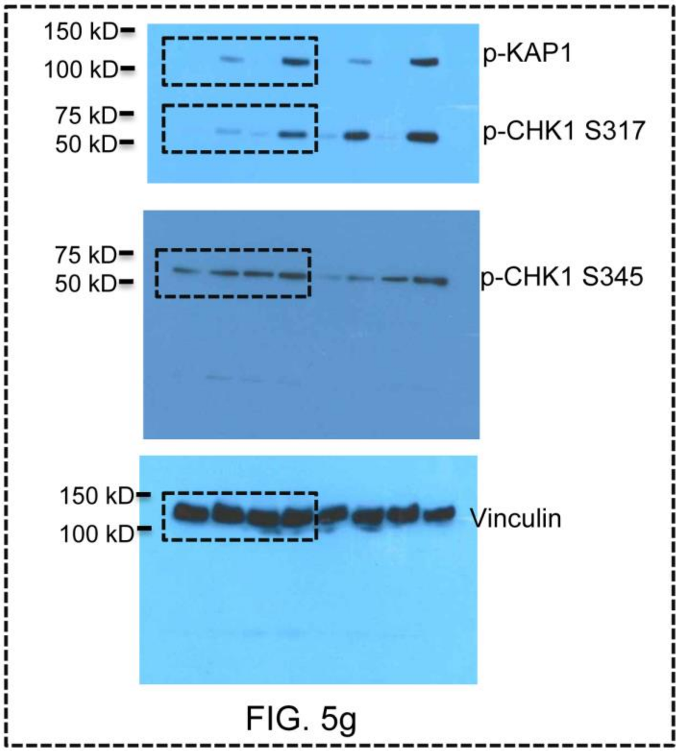

FIG. 5g

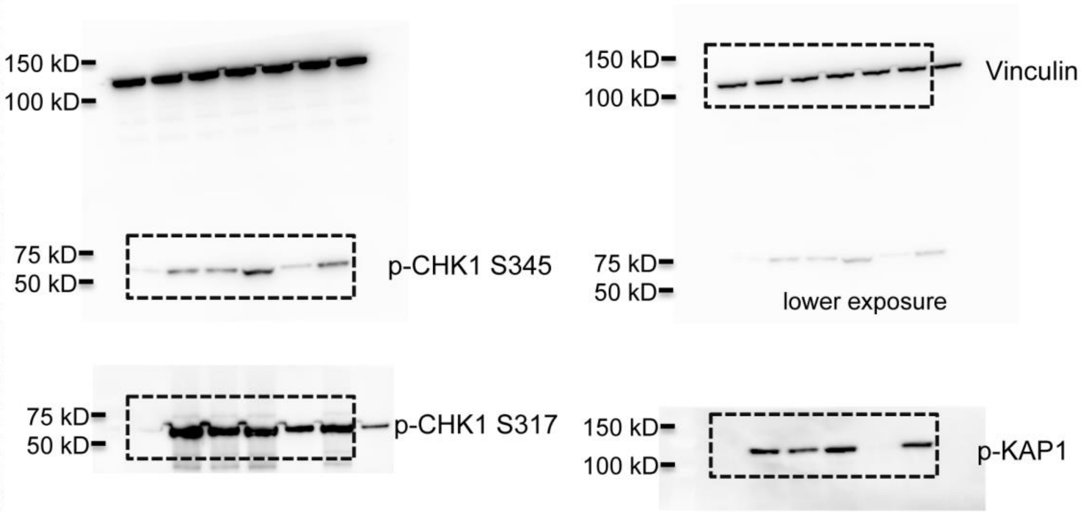
150 kD
100 kD
75 kD
50 kD
p-CHK1 S345
75 kD
50 kD
p-CHK1 S317
150 kD
100 kD
Vinculin
75 kD
50 kD
lower exposure
150 kD
100 kD
p-KAP1

FIG. 5h

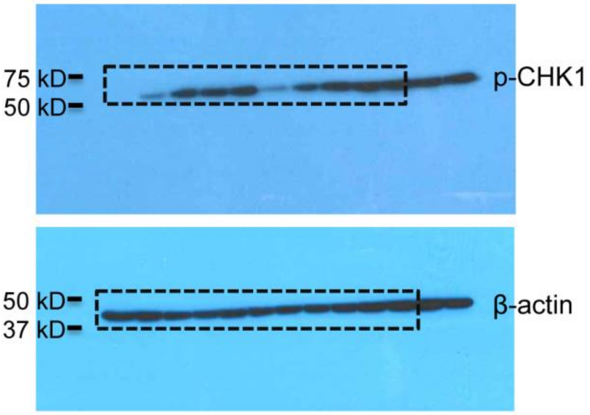
75 kD
50 kD
p-CHK1
50 kD
37 kD
β-actin

FIG. 6b

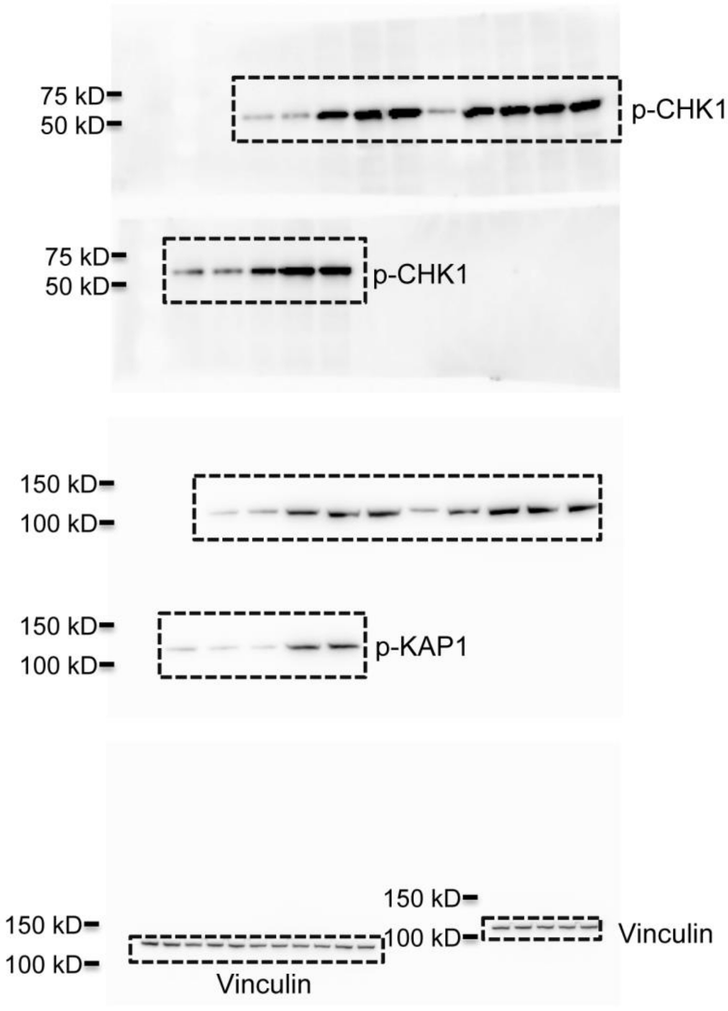

FIG. 6d

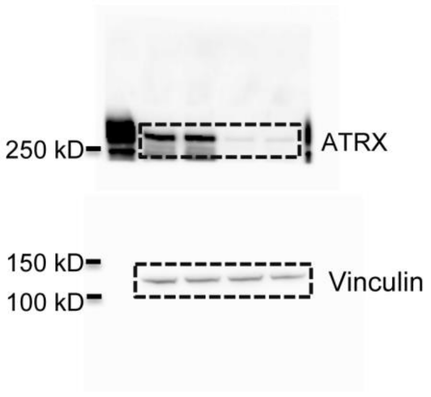

Supplementary FIG. 1e

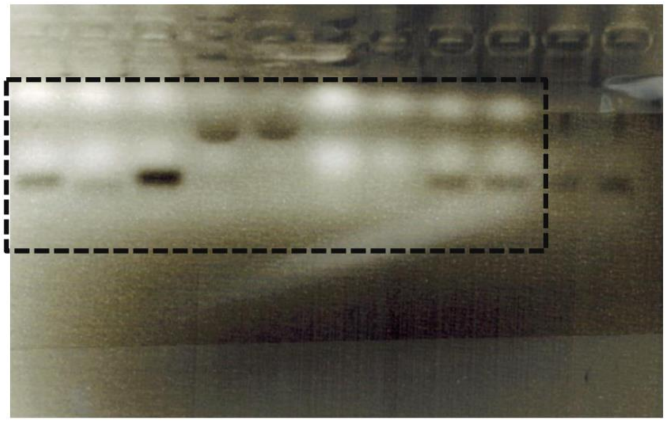

Supplementary FIG. 2a

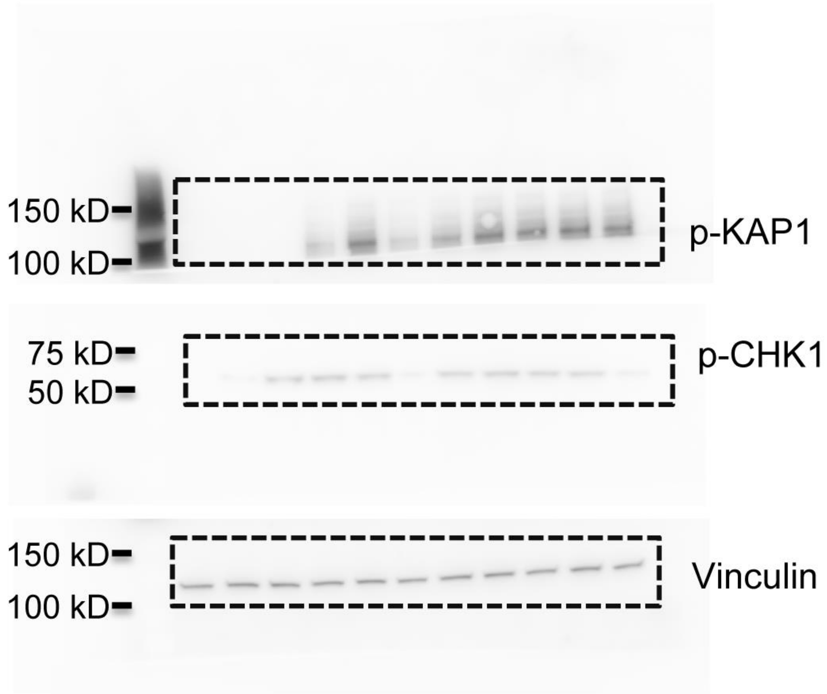

Supplementary FIG. 10b

Supplementary FIG. 12: Uncropped western blots used in figures. Molecular markers and paper figures associated with blots are indicated, as is the extent of horizontal cropping (p.c.: positive control).
