# Supplementary material for: G-quadruplex DNA drives genomic instability and represents a targetable molecular abnormality in ATRX-deficient malignant glioma
Source: Nat Commun. 2019 Feb 26;10:943. doi: 10.1038/s41467-019-08905-8 (PMC6391399; doi:10.1038/s41467-019-08905-8)
Supplement: Supplementary file 3 — Reporting Summary [file 41467_2019_8905_MOESM3_ESM.pdf]

nature research

Corresponding author(s): Jason T. Huse

Last updated by author(s): Jan 25, 2019

Reporting Summary

Nature Research wishes to improve the reproducibility of the work that we publish. This form provides structure for consistency and transparency in reporting. For further information on Nature Research policies, see Authors & Referees and the Editorial Policy Checklist.

Statistics

For all statistical analyses, confirm that the following items are present in the figure legend, table legend, main text, or Methods section.

n/a	Confirmed	
☐	☒	The exact sample size (*n*) for each experimental group/condition, given as a discrete number and unit of measurement
☐	☒	A statement on whether measurements were taken from distinct samples or whether the same sample was measured repeatedly
☐	☒	The statistical test(s) used AND whether they are one- or two-sided<br>*Only common tests should be described solely by name; describe more complex techniques in the Methods section.*
☒	☐	A description of all covariates tested
☒	☐	A description of any assumptions or corrections, such as tests of normality and adjustment for multiple comparisons
☐	☒	A full description of the statistical parameters including central tendency (e.g. means) or other basic estimates (e.g. regression coefficient) AND variation (e.g. standard deviation) or associated estimates of uncertainty (e.g. confidence intervals)
☐	☒	For null hypothesis testing, the test statistic (e.g. *F*, *t*, *r*) with confidence intervals, effect sizes, degrees of freedom and *P* value noted<br>*Give P values as exact values whenever suitable.*
☒	☐	For Bayesian analysis, information on the choice of priors and Markov chain Monte Carlo settings
☒	☐	For hierarchical and complex designs, identification of the appropriate level for tests and full reporting of outcomes
☐	☒	Estimates of effect sizes (e.g. Cohen's *d*, Pearson's *r*), indicating how they were calculated

Our web collection on statistics for biologists contains articles on many of the points above.

Software and code

Policy information about availability of computer code

Data collection	N/A
Data analysis	Raw data from Affymetrix Human SNP array 6.0 were processed by Partek Genome Suite 6.6, following its build-in copy number analyzing tool, to obtain the copy number data. The data were analyzed, visualized by GISTIC 2.0 and ggplot (R package) and presented on Figure 4 a-d and Supplementary Figure 5.

For manuscripts utilizing custom algorithms or software that are central to the research but not yet described in published literature, software must be made available to editors/reviewers. We strongly encourage code deposition in a community repository (e.g. GitHub). See the Nature Research guidelines for submitting code & software for further information.

Data

Policy information about availability of data

All manuscripts must include a data availability statement. This statement should provide the following information, where applicable:

- Accession codes, unique identifiers, or web links for publicly available datasets
- A list of figures that have associated raw data
- A description of any restrictions on data availability

All data (raw and processed) and materials related to this manuscript will be made available upon request, utilizing material transfer agreements when appropriate. Raw SNP array data and copy number variation profiles have been deposited in Gene Expression Omnibus (GSE125296). Raw western blot data is presented in Supplementary FIG. 12.

Field-specific reporting

Please select the one below that is the best fit for your research. If you are not sure, read the appropriate sections before making your selection.

☒ Life sciences ☐ Behavioural & social sciences ☐ Ecological, evolutionary & environmental sciences

For a reference copy of the document with all sections, see nature.com/documents/nr-reporting-summary-flat.pdf

Life sciences study design

All studies must disclose on these points even when the disclosure is negative.

Sample size	No sample-size calculations were performed. Sample size was determined to be adequate based on the magnitude and consistency of measurable differences between groups.
Data exclusions	On principle, data were only excluded for failed experiments, reasons for which included microbial contamination and failed positive/negative controls.
Replication	Replicate experiments were successful.
Randomization	Mice were randomized whenever possible.
Blinding	Investigators were not blinded in this study regarding treatment versus vehicle arms. However, experimental metrics were quantitative.

Reporting for specific materials, systems and methods

We require information from authors about some types of materials, experimental systems and methods used in many studies. Here, indicate whether each material, system or method listed is relevant to your study. If you are not sure if a list item applies to your research, read the appropriate section before selecting a response.

Materials & experimental systems

n/a	Involved in the study	
☐	☒	Antibodies
☐	☒	Eukaryotic cell lines
☒	☐	Palaeontology
☐	☒	Animals and other organisms
☒	☐	Human research participants
☒	☐	Clinical data

Methods

n/a	Involved in the study	
☒	☐	ChIP-seq
☒	☐	Flow cytometry
☒	☐	MRI-based neuroimaging

Antibodies

Antibodies used	Information provided in Supplementary Table 1.
Validation	All antibodies, except 1H6 for G-quadruplex, were purchased from commercial source, with the validation information is available on their websites. For 1H6 antibody, DNase and RNase treated cells were performed to validate the specificity to DNA G-quadruplex. The specificity of 1H6 is also supported by previously published articles (e.g.: Nucleic Acids Res. 42(2): 860–869., J. Biol. Chem., 291: 18041–18057).

Eukaryotic cell lines

Policy information about cell lines

Cell line source(s)	Information of source of cell lines is provided in the manuscript, either in the "Results" or "Methods" section.
Authentication	Cell lines used in this study were routinely monitored for morphology and specific protein expression.
Mycoplasma contamination	All cell lines used in this study were regularly tested for mycoplasma contamination at the Antibody and Bioresource Core of MSKCC every 3 months.
Commonly misidentified lines (See ICLAC register)	No commonly misidentified lines were used in this study.

Animals and other organisms

Policy information about studies involving animals; ARRIVE guidelines recommended for reporting animal research

Laboratory animals	Mouse, Nude (Stock #: NCRNU, TACONIC FARMS INC.), Female, 5-6 weeks, were used in this study.
Wild animals	N/A
Field-collected samples	N/A
Ethics oversight	This study was approved and overseen by the MD Anderson Cancer Center and Memorial Sloan-Kettering Cancer Center IBC and IACUC committees.

Note that full information on the approval of the study protocol must also be provided in the manuscript.
